# A Rare Case of Retroperitoneal Tumefactive Fibroinflammatory Lesion Related to IgG4-Sclerosing Disease

**DOI:** 10.7759/cureus.61968

**Published:** 2024-06-08

**Authors:** Spyridon Nikas, Sotiria Zonitsa, Pavlos Anastasiadis, Kalliopi Veniadou, Sofia Roumelioti, Angelos C Mitsas, Georgios Gerasopoulos, Panagiotis Gkouvis

**Affiliations:** 1 Radiology Department, General Hospital of Imathia, Veria Unit, Veria, GRC; 2 Surgical Department, General Hospital of Imathia, Veria Unit, Veria, GRC

**Keywords:** retroperitoneal mass, retroperitoneum, fibroinflammatory tumor, tumefactive lesions, igg4-related disease

## Abstract

We present a unique case of a retroperitoneal tumefactive fibroinflammatory lesion related to IgG4-sclerosing disease; it is a rare manifestation of the IgG4-related disease, which usually causes diffuse fibrosis when located in the retroperitoneum, rather than mass-like lesions. A 49-year-old man presented to the emergency department complaining of abdominal pain and vomiting. Subsequent testing with abdominal ultrasound, CT, and MRI revealed a large retroperitoneal mass of unknown origin, heterogenous, with a concentric circles pattern best visualized in MRI. The lesion was resected, and the histological and immunohistochemical studies revealed an IgG4-related tumefactive fibroinflammatory lesion of the retroperitoneum.

## Introduction

Retroperitoneal masses are rare but potentially lethal entities, with a very heterogenous origin. They are either primary or secondary and mostly malignant, with lymphomas accounting for about one-third of all primary retroperitoneal masses. Sarcomas also represent about one-third of all primary retroperitoneal masses, with liposarcomas being the most common variant by far [1-3]. They remain mostly asymptomatic, either until they present as palpable masses or until their space-occupying and sometimes infiltrating behavior gives rise to symptoms attributed to the organs compressed or infiltrated (i.e. small bowel obstruction, hydronephrosis), by which time most of these lesions are already very large and complete resection is more difficult and the prognosis is generally poor [3-5]. There are also several benign lesions related to the retroperitoneum, such as extramedullary hemopoiesis and schwannomas, that may mimic malignant neoplasms and need to be distinguished from those since the operative risk associated with retroperitoneal mass excision is relatively higher.

IgG4-related disease is a poorly understood multisystemic disease that can affect virtually every organ or system in the human body and tends to form tumefactive masses [6]. It is related to several autoimmune diseases, such as autoimmune pancreatitis and Hashimoto thyroiditis. Even though its exact pathogenetic mechanism has not been established yet, it is thought to be immune-mediated [6,7]. When located in the retroperitoneum, IgG4-related disease tends to manifest as diffuse fibrotic changes, frequently leading to imaging findings such as diffuse soft-tissue deposits around the great retroperitoneal vessels, mostly periaortic, whereas extension to the renal pelvis and hydronephrosis is relatively common [8,9].

The manifestation of the disease as a focal tumor-like lesion in the retroperitoneum is very rare, and it is very difficult to differentiate it from lesions such as a solitary fibrous tumor (SFT) or an inflammatory myofibroblastic tumor (IMT) preoperatively as imaging characteristics of such masses are virtually unknown [10,11]. Cross-sectional imaging is frequently employed in the diagnostic process of patients with suspected retroperitoneal pathology. While CT and MRI are very sensitive in detecting these lesions, they are not always very specific, especially regarding solid tumors [2,12,13]. A CT-guided biopsy is an important tool in these cases; however, the final, definitive diagnosis is usually provided by histological and immunohistochemical studies after surgical excision of the lesion. Even then, a definitive answer may remain elusive. We present a rare case of a tumefactive fibroinflammatory lesion of the retroperitoneum related to IgG4-sclerosing disease, which was initially misdiagnosed as malignancy. We describe its imaging characteristics across several imaging modalities and explore its histologic and immunohistochemical features.

This case report was previously presented as an e-poster at the 26th Panhellenic Congress of Radiology.

## Case presentation

Patient information, clinical findings, and laboratory tests

A 49-year-old Caucasian male construction worker presented to the emergency department of a community hospital with complaints of colicky, mild abdominal pain during the previous month. The patient also complained of vomiting during the past few hours, with normal bowel movements. He also mentioned recurrent back pain in the past six months. No relevant medical, surgical, family, or psychosocial history was noted. The patient’s general appearance was healthy, and the physical examination revealed only a mildly tender abdomen around the umbilicus and to the left, without any signs of distention or peritonism. The laboratory test results were unremarkable. The patient was further submitted to imaging tests.

Imaging studies

**Figure 1:**
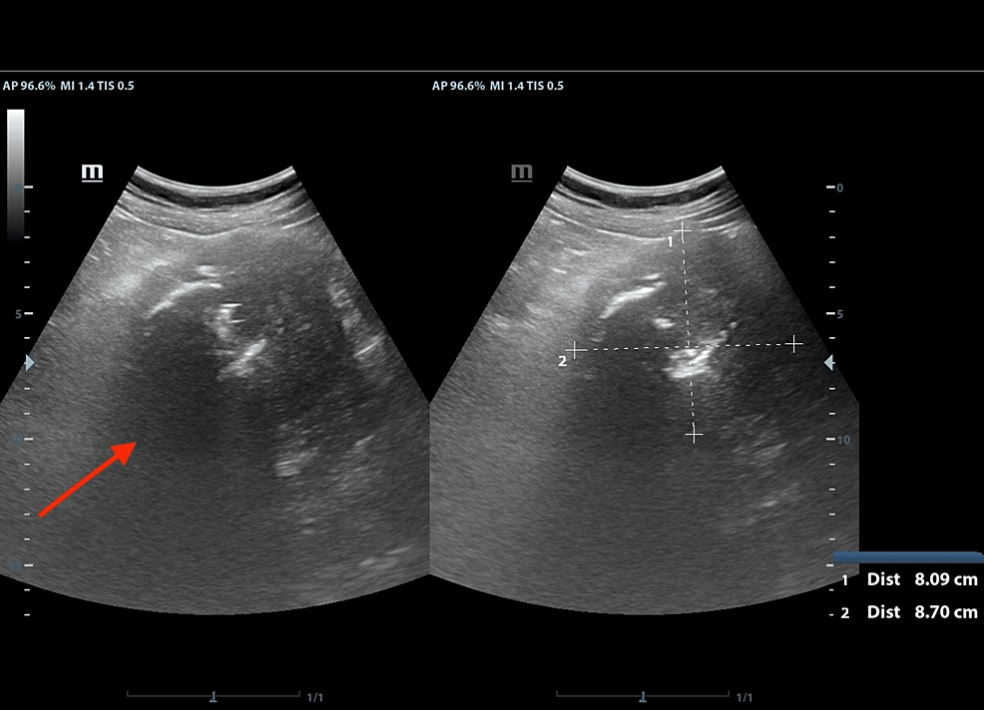
Ultrasound appearance of the lesion Note the gross calcifications and the heterogeneity of the lesion. The deep borders of the mass are indiscernible

The patient underwent an abdominal ultrasound as part of his workup, which showed a large heterogenous mass between the left kidney and the pancreatic tail, with gross calcifications in it (Figure 1).

**Figure 2:**
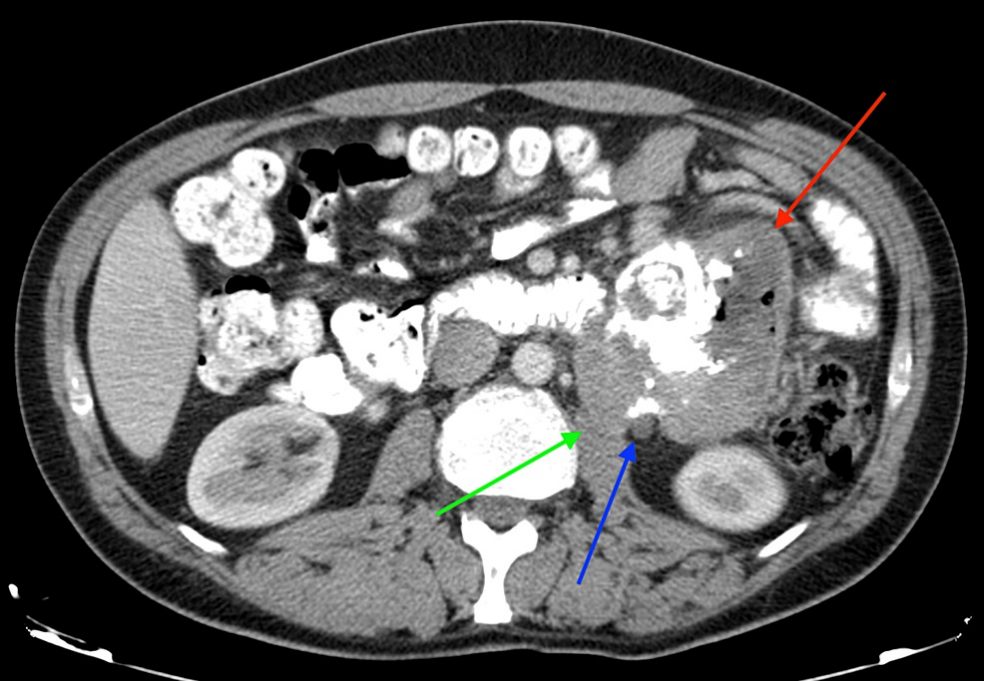
Axial CT appearance of the retroperitoneal mass The red arrow shows the lesion, which displays central foci of calcification, air, and areas of necrosis. The green arrow shows the left psoas muscle infiltration, and the blue arrow shows the strangulation of the left ureter by the lesion in the retroperitoneal space CT: computed tomography

**Figure 3:**
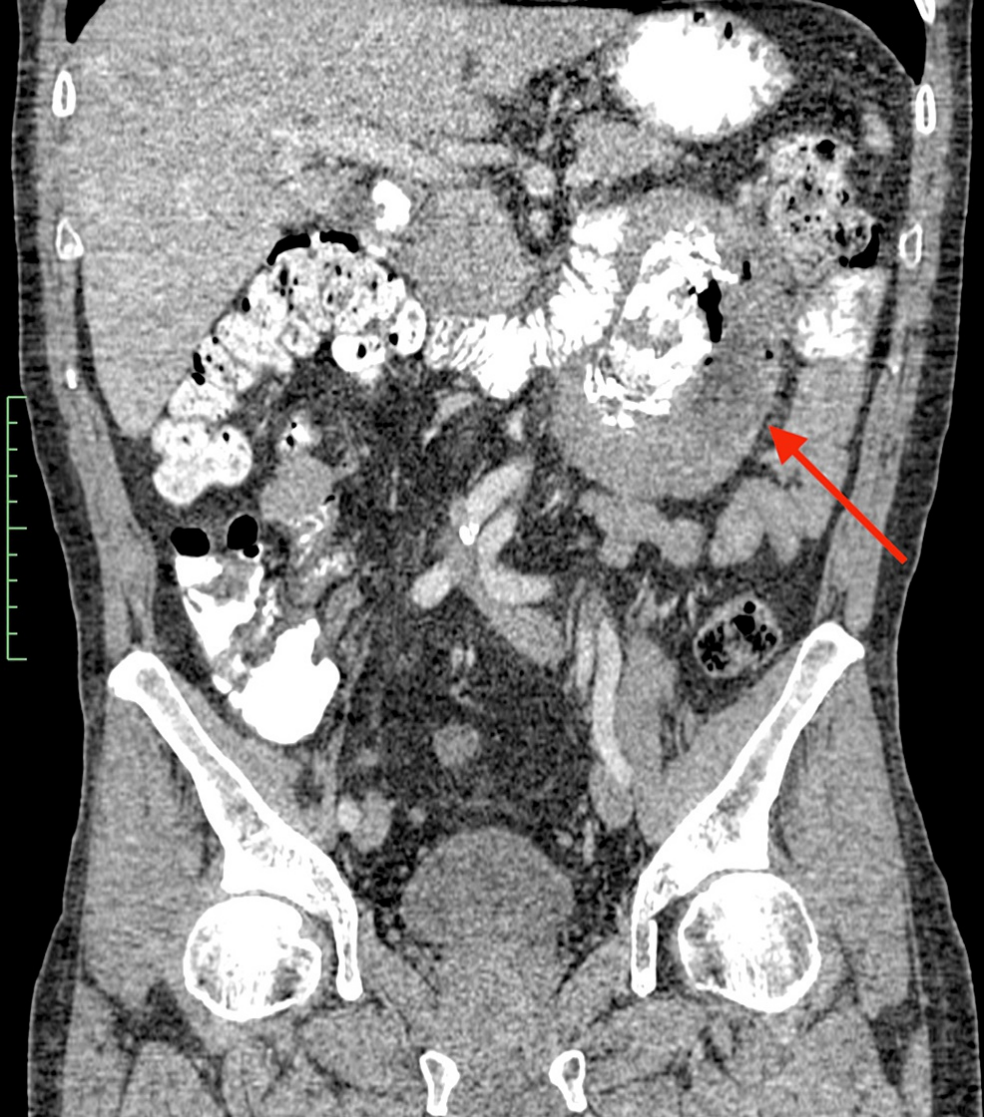
Coronal CT appearance of the retroperitoneal mass Central rings of calcification, with foci of air, are noted, probably due to direct infiltration of adjacent bowel loops CT: computed tomography

A CT of the abdomen and pelvis with intravenous and oral contrast revealed a large heterogenous mass of ~12 cm maximum diameter emerging in the retroperitoneal space. The lesion showed a plethora of characteristics, with multiple different soft tissue densities, gross calcification areas and even air bubbles, mild contrast uptake areas, and necrotic foci, and it also demonstrated an aggressive behavior with infiltration of the adjacent jejunal loops and the descending colon, as well as infiltration of the left psoas muscle. Further infiltration of the left renal hilum was noted, with strangulation but not infiltration of the renal vessels and the left ureter. No lymphadenopathy was noted (Figures 2-3).

**Figure 4:**
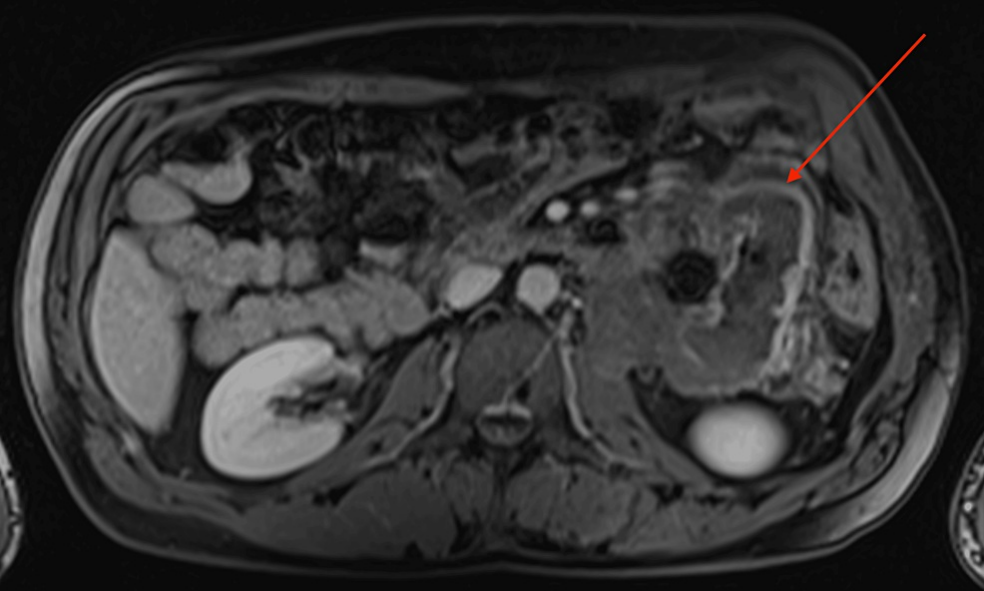
Axial MRI T1-weighted image, with intravenous contrast infusion Note the heterogeneity, the infiltration of the left psoas muscle, and the peripheral signal enhancement after intravenous contrast infusion MRI: magnetic resonance imaging

**Figure 5:**
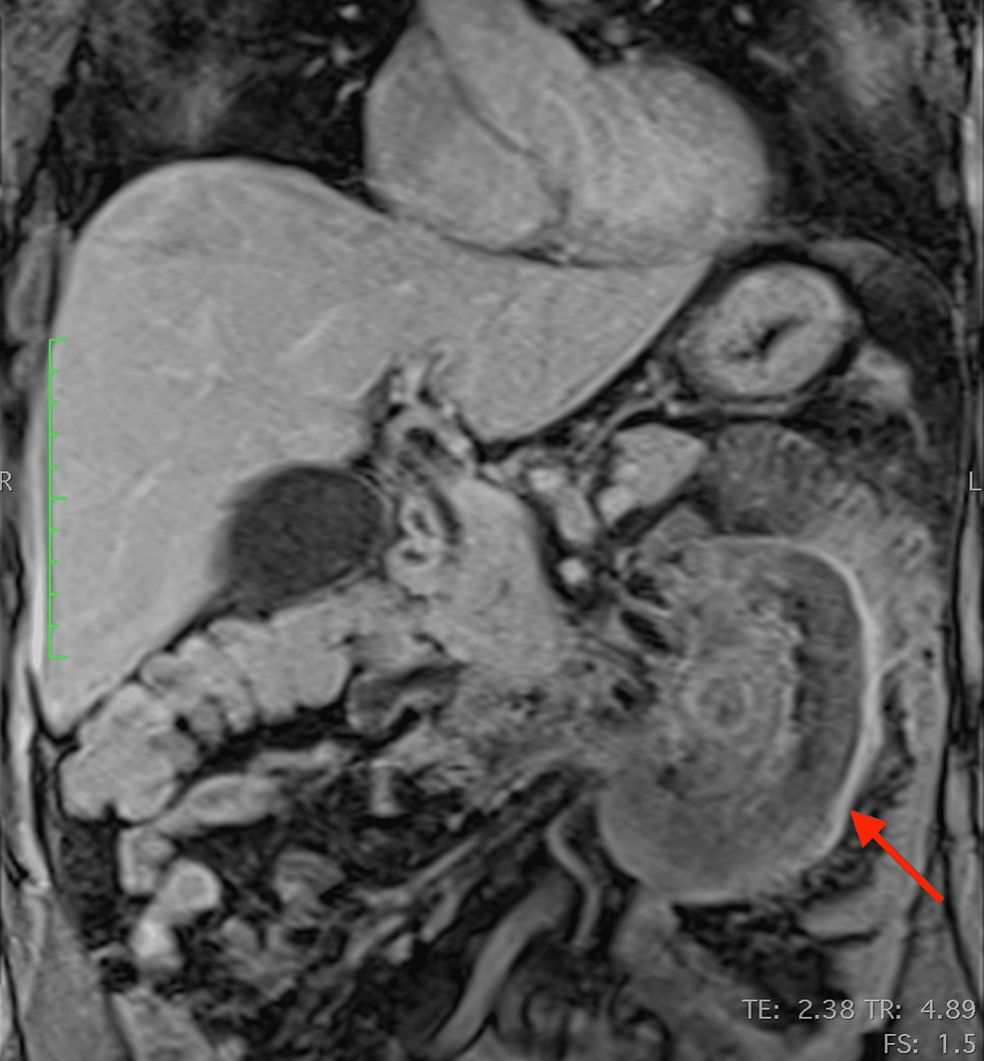
Coronal MRI T1-weighted image, with intravenous contrast infusion Note the peripheral signal enhancement after intravenous contrast infusion MRI: magnetic resonance imaging

**Figure 6:**
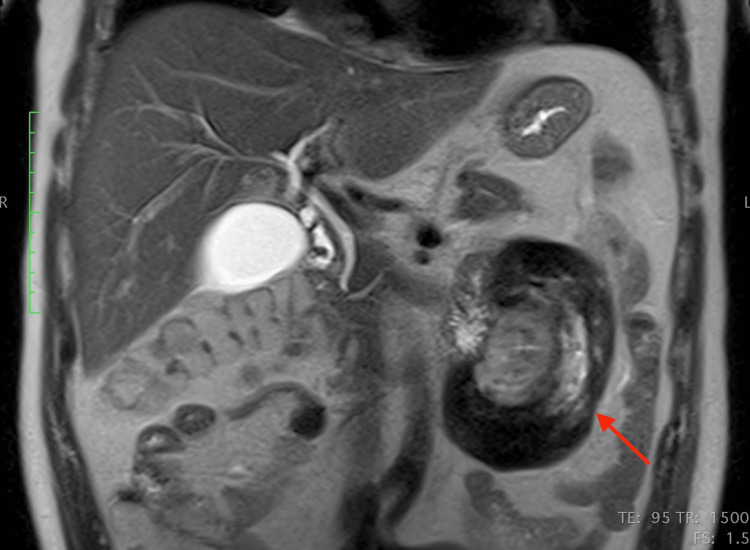
Coronal MRI T2-weighted image MRI: magnetic resonance imaging

**Figure 7:**
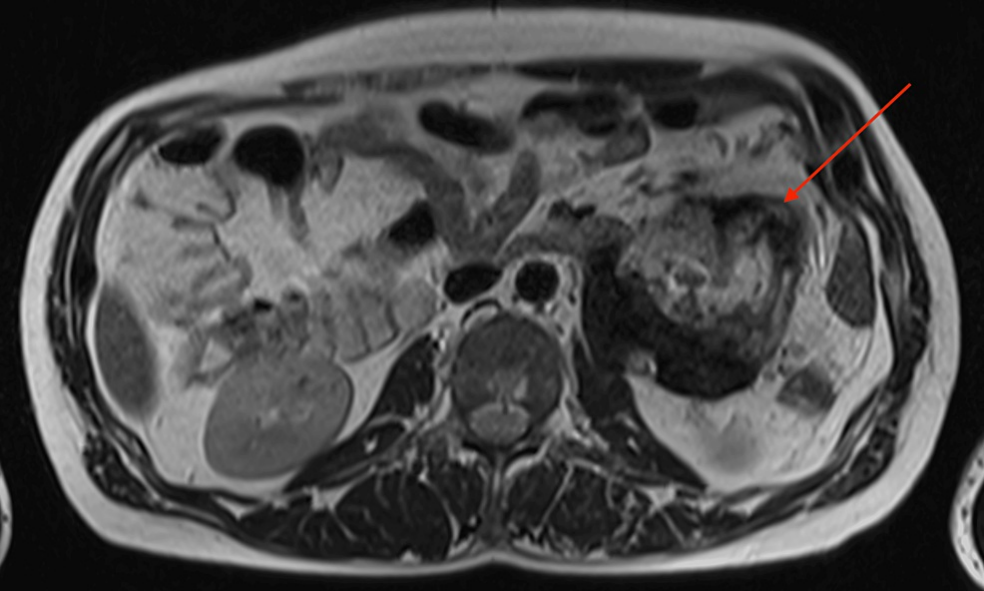
Axial MRI T2-weighted image MRI: magnetic resonance imaging

**Figure 8:**
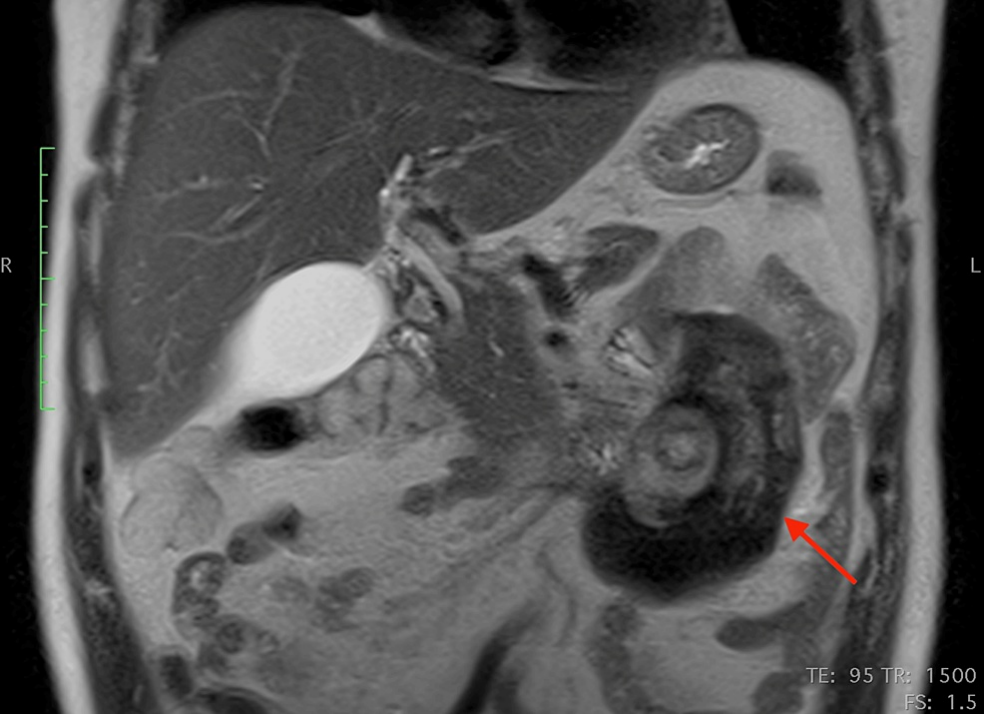
Coronal MRI T2-weighted image (different level compared to Figure 6) The concentric circles-like pattern (like “onion layers”) is best appreciated in the T2 images MRI: magnetic resonance imaging

The patient further underwent an MRI to characterize the lesion. The mass was well-defined and heterogeneous in T2 sequences, with low signal peripherally and intermediate signal centrally, whereas a peripheral contrast uptake was noted in T1 sequences with intravenous contrast infusion. Infiltration of adjacent structures, as seen in the CT, was confirmed. The most notable characteristic was the pattern of concentric circles of different, alternating signal intensities within the mass, best seen in T2 sequences, with an enhancing capsule. (Figures 4-8).

Diagnosis and treatment

**Figure 9:**
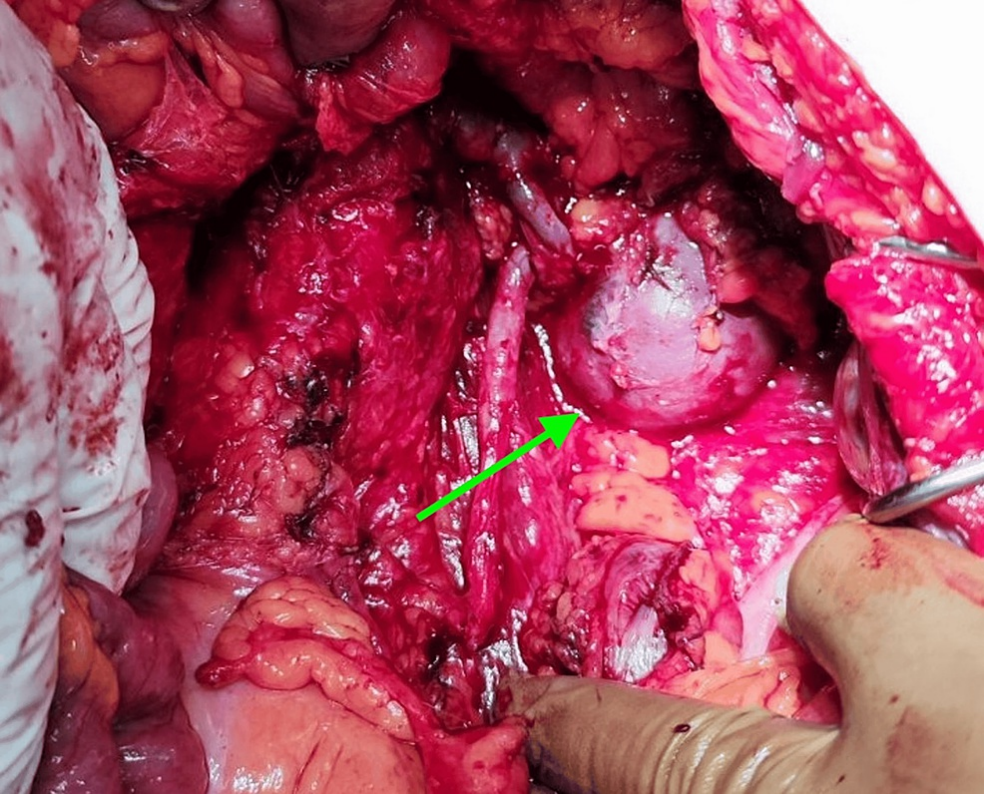
Intraoperative photograph of the lesion Note the bulging of the mass in the retroperitoneal space, next to the large vessels of the abdomen

A provisional diagnosis of a sarcoma-like tumor was made, and the patient was operated on. On the tenth day of admission, the patient was led to the operating room, where the retroperitoneal cavity was exposed through a wide midline incision. The lesion was fully excised en-bloc along with the fourth part of the duodenum, the Treitz ligament, part of the mesentery, around 30 cm of the jejunum, and the descending colon. The tail of the pancreas was spared, as was the left kidney, where the tumor was encasing but not infiltrating the left renal vessels and the left ureter. The intestinal continuity was restored utilizing a Roux-en-Y gastroenteric anastomosis and a side-to-side colocolic anastomosis. The retroperitoneal and peritoneal cavities were thoroughly irrigated, and three vacuum drains were placed. On the fourth postoperative day, the nasogastric feeding tube was removed followed by the the surgical drains on the fifth postoperative day. The patient showed a gradual clinical and laboratory improvement, and he was discharged uneventfully on the eighth postoperative day. Figure 9 shows an intraoperative photograph of the lesion located in the retroperitoneum.

The histological study revealed extensive chronic inflammatory changes, with clusters of lymphocytes and plasma cells between collagen fibers, storiform fibrosis, and vessel wall infiltration from these cells and small fat necrosis areas. As for immunohistochemical studies, the anaplastic lymphoma kinase (ALK) marker was negative, as were the markers desmin, CD34, and β-catenin. The Ki67 marker was lower than 2%. Further immunohistochemical studies revealed very high IgG plasma cells, with an elevated number of IgG4 cells that exceeded 35 and even 40 cells per high-power field, with an IgG4/IgG ratio >30%. Thus, a working diagnosis of an IgG4-related tumefactive fibroinflammatory lesion of the retroperitoneum was made, with the primary differential diagnosis being the inflammatory myofibroblastic tumor of the retroperitoneum [14]. No signs of malignancy were found. One year postoperatively, the patient is doing well, and no recurrence has been noted in follow-up imaging tests. The patient was referred to an external rheumatologist for further evaluation. Unfortunately, subsequent external laboratory testing was not available.

## Discussion

This study aims to raise awareness of yet another great mimicker of malignancy in the retroperitoneum, the tumefactive fibroinflammatory lesion related to IgG4-sclerosing disease. This is a unique manifestation of the IgG4-related disease because such tumefactive lesions are extremely rare in the retroperitoneum, where it is more common to encounter fibrotic changes [5,6]. Solid retroperitoneal masses are difficult to diagnose based solely on imaging studies. Our case displayed gross calcifications, which are observed in several sarcomas [dedifferentiated liposarcoma, undifferentiated pleomorphic sarcoma, teratoma, malignant fibrous histiocytoma, and extragastrointestinal stromal tumor (EGIST)] as well as intratumoral necrosis, which is a sign of malignancy and aggressiveness, also compatible with sarcomas [15]. Due to these findings, the lesion was misdiagnosed as some kind of sarcoma.

This case, apart from the above non-specific signs, further demonstrated a very interesting imaging feature: concentric circles of different density and signal intensity in CT and MRI respectively, best appreciated in MRI T2 sequences. One probable theory that could explain this pattern is that these concentric circles represent different stages of chronic inflammation, corresponding to inflammatory flare-ups of the disease. Every inflammatory flare would produce yet another circle (like an onion layer) and this would also explain the significant differences in the appearance of every “ring”. This could explain the patient's recurrent back pain. However, this is a "post hoc" theory and should be considered with a grain of salt.

This case study has several limitations. Since our institution is a relatively small community hospital, there were limitations related to clinical practice; the patient did not undergo a CT-guided biopsy before surgical excision, and the MRI was performed in an associated external laboratory, that did not acquire diffusion-weighted imaging (DWI) sequences, which could have provided valuable information regarding the nature of this entity. Another limitation is the unavailability of subsequent laboratory testing necessary for the work-up of IgG4-related disease since the patient was referred to an external rheumatologist after the histological and immunohistochemical diagnosis was made. Nevertheless, this case is rare and, to the best of our knowledge, unique in terms of its manifestation and imaging appearance. Reports of more such cases are needed to expand our scope of knowledge regarding this condition.

## Conclusions

The tumefactive fibroinflammatory lesion related to IgG4-sclerosing disease is a rare manifestation of IgG4-related disease. Since its imaging characteristics are scarce, radiologists should be aware of this presentation of the disease and correlate findings such as retroperitoneal tumors with laboratory studies.
